# Metabolic and nutritional support of critically ill patients: consensus and controversies

**DOI:** 10.1186/s13054-015-0737-8

**Published:** 2015-01-29

**Authors:** Jean-Charles Preiser, Arthur RH van Zanten, Mette M Berger, Gianni Biolo, Michael P Casaer, Gordon S Doig, Richard D Griffiths, Daren K Heyland, Michael Hiesmayr, Gaetano Iapichino, Alessandro Laviano, Claude Pichard, Pierre Singer, Greet Van den Berghe, Jan Wernerman, Paul Wischmeyer, Jean-Louis Vincent

**Affiliations:** Department of Intensive Care, Erasme University Hospital, Université Libre de Bruxelles, 808 route de Lennik, Brussels, 1070 Belgium; Department of Intensive Care, Gelderse Vallei Hospital, Willy Brandtlaan 10, Ede, Gld 6716RP The Netherlands; Service de Médecine Intensive Adulte et Brûlés, CHUV BH 08.612, Lausanne, CH 1011 Switzerland; Department of Medical, Surgical and Health Sciences, Clinica Medica AOUTS, University of Trieste, via Farneto 3, Trieste, 34142 Italy; Department and Laboratory of Intensive Care Medicine, University Hospitals Leuven (UZ Leuven), Herestraat 49, Leuven, B-3000 Belgium; Northern Clinical School Intensive Care Research Unit, University of Sydney, Reserve Road, St Leonards, NSW 2065 Australia; Department of Medicine, University of Liverpool, Liverpool Merseyside, L69 3BX UK; Clinical Evaluation Research Unit, Kingston General Hospital, Kingston, ON K7L 2 V7 Canada; Division of Cardiac-Thoracic-Vascular Anesthesia and Intensive Care, Medical University Vienna, Spitalgasse 23, Wien, 1090 Austria; Department of Anesthesiology and Intensive Care, Universita’ degli Studi di Milano, via Di Rudini’ 8, Milano, 20142 Italy; Department of Clinical Medicine, Sapienza University, Piazzale Aldo Moro 5, Roma, 00185 Italy; Department of Nutrition, Geneva University Hospital, Rue Gabrielle-Perret-Gentil 4, Geneva, 1211 Switzerland; Department of Intensive Care, Beilison Hospital, Petah Tikva, 49100 Israel; Department of Anesthesiology & Intensive Care Medicine, Karolinska University Hospital, Huddinge, Stockholm, 141 86 Sweden; Department of Anesthesiology, University of Colorado School of Medicine, 12700 E. 19th Ave, Box 8602, Aurora, CO RC2 P15-7120 USA

## Abstract

The results of recent large-scale clinical trials have led us to review our understanding of the metabolic response to stress and the most appropriate means of managing nutrition in critically ill patients. This review presents an update in this field, identifying and discussing a number of areas for which consensus has been reached and others where controversy remains and presenting areas for future research. We discuss optimal calorie and protein intake, the incidence and management of re-feeding syndrome, the role of gastric residual volume monitoring, the place of supplemental parenteral nutrition when enteral feeding is deemed insufficient, the role of indirect calorimetry, and potential indications for several pharmaconutrients.

## Introduction

Nutritional support in the acutely ill is a complex subject. Several recent studies have led to considerable changes in our understanding of the metabolic response to critical illness and of various aspects of nutritional management, including monitoring of the metabolic response and the determination of caloric, protein, and micronutrient requirements. The aims of this review are to summarize recent findings, to highlight areas of consensus and controversy, and to define priorities for further research.

## Metabolic response, inflammation, and anabolic resistance

The metabolic response to stress is part of the adaptive response to survive acute illness. During stress, several mechanisms that have been well preserved through evolution are triggered to increase the provision of energy substrates to vital tissues, including stimulation of the sympathetic nervous system, release of pituitary hormones [1], and peripheral resistance to the effects of anabolic factors [2]. Recent findings suggest that hormones released from the gut and adipose tissue may be involved as additional triggers of the response to stress and critical illness [3]. As a result of this complex metabolic response, the control of energy substrate utilization is only partially regulated by substrate availability. Instead, pathways of energy production are altered and alternative substrates can be used. Clinically, one can identify a variety of changes, including increased energy expenditure (EE), stress hyperglycemia, loss of muscle mass, and eventually psychological and behavioral problems [4,5].

The role of inflammation in the metabolic response to stress has been recognized for a long time and is currently under increased scrutiny after the results of the trials from Leuven University [6,7], in which the magnitude of the inflammatory response was attenuated in patients who received intensive insulin therapy (IIT) [6] and increased in patients who received no parenteral nutrition during the first week of critical illness [7]. Experimental findings [8,9] have consistently indicated that high glucose concentrations increase the production or expression (or both) of pro-inflammatory mediators, adherence of leukocytes, and alterations in endothelial integrity and decrease chemotaxis and phagocytic activity and release of reactive oxygen species (ROS) by neutrophils, whereas insulin exerts the opposite effects [10]. High doses of insulin were found to reduce levels of C-reactive protein in critically ill patients [11,12], and interleukin-6, interleukin-8, and tumor necrosis factor levels in patients on extracorporeal circulation [13] or with burns [14]. The expression of adhesion molecules on the endothelium was reduced as was the transcription of inducible nitric oxide (NO) synthase gene in the liver and muscle of patients randomly assigned to IIT [15]. These effects in patients treated with IIT could be related to the anti-inflammatory effects of insulin or to an attenuation of the pro-inflammatory effects of hyperglycemia or both [16]. The available clinical data suggest that prevention of severe hyperglycemia may reduce cell damage; however, preventing hyperglycemia by using high doses of insulin, as required in cases of high intake of carbohydrates, can blunt the early inflammatory response.

Resistance to the anabolic signals leading to loss of muscle protein and function is a major long-term consequence of stress metabolism [17]. An infusion of amino acids in healthy volunteers rapidly increases the rate of muscle protein synthesis [18], whereas in critically ill patients the rate of protein degradation increases more than the rate of protein synthesis, resulting in a negative muscle protein balance [19]. Kinetic studies have demonstrated an impairment in the amino acid transport systems and increased shunting of blood away from the muscles [20]. The underlying mechanisms have been partially unraveled and include a relative resistance to insulin [21], which is further amplified by physical inactivity [22,23]. In theory, omega-3 fatty acids, pentoxifylline, growth hormone, testosterone, and beta blockade could also preserve muscle strength and dampen protein catabolism [2] and thereby help to prevent the long-term muscular consequences of the metabolic response to stress.

Monitoring the metabolic response is a major clinical challenge because it relies on non-specific clinical and biochemical markers: secondary infections, muscle atrophy and weakness, respiratory insufficiency, delayed wound healing, and a high incidence of secondary complications indicate prolonged catabolism; in contrast, severe hyperglycemia, liver steatosis, respiratory insufficiency with severe hypercapnia, and immune depression, again leading to increased infectious complications [24], can be related to overfeeding [25]. Recently, metabolomic profiling of body fluid was reported as a promising approach to better characterize the metabolic derangements of critical illness [26,27].

## Nutritional requirements

It is difficult to predict EE in the critically ill as predictive equations fail to match measured EE in about 80% of patients [28], and protein losses cannot be estimated without specific measurement. Most studies have reported a high incidence of unintentional underfeeding (that is, a lower actual caloric and protein intake than the amount prescribed). An association between the amount of calories prescribed and several outcome variables has been reported by several groups of investigators [29-32]. Similarly, positive associations between protein intake and survival have been reported in observational data collections [33,34]. A major weakness of these observational studies relates to the heterogeneity in the severity of illness, a key potential confounder; less sick patients tolerate enteral nutrition better, are more adequately fed, and have better outcomes. Moreover, these findings may be related to informative censoring [33], and unequivocal confirmation by other recent robust trials is still awaited [35,36]. Nevertheless, the optimal intake of macronutrients is largely undefined, and results of the prospective trials discussed below have given controversial results. This uncertainty is partly related to the lack of accurate monitoring tools. Computerized information systems may help prevent under- and overfeeding [37].

Although the effects of energy and proteins are intertwined, we discuss caloric and protein requirements separately. Ideally, future clinical trials should assess the effects of changes in the intakes of only calories or only proteins. Likewise, the effects of energy source (carbohydrates or fat) should be studied in adequately powered prospective trials.

### Energy requirements

What represents optimal energy intake in critically ill patients and whether caloric intake should match resting EE are hot topics of debate [38,39]. However, the assessment of EE in the critically ill is a major challenge [28,40], even when using predictive equations, and can lead to over- or underfeeding especially as EE may be elevated and can vary over time. Moreover, predictive equations are not sufficiently accurate for reliable use in critically ill patients [28]. Nevertheless, measurement of EE is feasible using indirect calorimetry, and guidelines from both the European Society for Clinical Nutrition and Metabolism [41] and the American Society for Parenteral and Enteral Nutrition [42] recommend use of this technique, although the accuracy of different indirect calorimeters has recently been challenged [43,44]. An association between the amount of calories prescribed and several outcome variables (for example, 2-month mortality, length of stay, and rate of complications) has been reported by several groups of investigators [29-32]. A large multicenter observational study in mechanically ventilated patients defined the optimal amount of intake as 80% of that which was prescribed [32]. Likewise, the Tight Calorie Control Study pilot study [24] reported improved hospital survival in a per-protocol analysis of the group of patients in whom caloric intake matched the measured EE; intention-to-treat analysis, however, revealed no survival benefit and moreover showed increased ICU length of stay and duration of mechanical ventilation together with a higher incidence of infections in this group. EE should probably be matched by caloric intake after the early phase of critical illness, but the proportion of the measured EE that should be administered likely varies over time.

The rationale for adequately matching caloric intake with caloric expenditure lies in the accelerated muscle catabolism that occurs when caloric supply is restricted, especially in patients confined to bed rest [45] and on the associations between caloric debt and poor outcome [29-31]. Arguments against the matching of calorie intake to EE during the early phase of critical illness include physiological evidence (that is, continuous endogenous production of glucose matching 50% to 75% of EE for the first few days after injury) and the suppression of autophagy by exogenous macronutrients. However, macronutrients can exert different effects on autophagy [46]. In particular, the inhibitory role of protein on autophagy has been reported [47] and could have contributed to the findings of worse outcome in the early parenteral nutrition group of the Impact of Early Parenteral Nutrition Completing Enteral Nutrition in Adult Critically Ill Patients (EPaNIC) study [48]. Moreover, the results of other prospective interventional trials have consistently shown either increased morbidity when caloric supply was increased [7,24] or no immediate benefit associated with supplemental parenteral nutrition in patients intolerant to early enteral nutrition (EEN) during the first 3 days of ICU admission [49]. Other recent interventional studies [50-53] were unable to show an improvement in outcome following an increase in caloric and protein intake. Of note, these trials were not designed or powered as equivalence studies and do not provide definitive data to inform clinicians about how much nutritional support is enough [54]. However, a *post hoc* analysis of the EPaNIC trial suggested that the smallest amount of nutrients was associated with the fastest recovery, and any higher dose was associated with a delay in recovery [55]. Moreover, this observational study tackled the issue of duration of ICU stay being associated with a higher likelihood of additional complications and higher amounts of nutritional intake by analyzing nutrition given over identical time spans of 3, 5, 7, 10, and 14 days. The findings related to early supplemental parenteral nutrition should not discourage attempts to optimize energy delivery by the enteral route [56], even though it was not associated with clinical benefit, or the need to identify patients at high mortality risk due to pre-ICU malnutrition [57].

### Protein requirements

The issue of optimal protein intake is no simpler than that of caloric intake. Essentially, the pool of free amino acids is fueled by the degradation products of tissue proteins, *de novo* synthesized amino acids, and nutritional intake. These amino acids are incorporated into proteins, involved in the regulation of specific pathways, or oxidized and removed as urea. The minimal protein requirement can be defined as the amount required to maintain a neutral tissue protein balance, at least in physiological conditions [58]. During critical illness, however, the breakdown of proteins is markedly increased and the types of protein synthesized differ considerably from healthy conditions. Recently, Rooyackers and colleagues [59] demonstrated that protein synthesis was markedly increased in patients with multiple organ failure. In addition, several pathways potentially regulated by amino acids are activated, and the mechanisms of clearance, including renal function, are often impaired. Therefore, the optimal amount of protein in critically ill patients cannot be deduced from data recorded in healthy subjects.

In critical illness, the loss of lean body mass, together with physical inactivity, is associated with increased proteolysis via the proteasome/ubiquitin pathway [60]. These findings generated the hypothesis that increased protein requirements are related to (a) the need for a greater amount of amino acid to achieve the same muscular synthesis rate, as a result of the anabolic resistance; (b) the need for amino acids for the synthesis of acute-phase response proteins; (c) the need for cysteine, the rate-limiting step of glutathione synthesis, in order to limit oxidative stress [61]; and (d) the prevention of glutamine depletion in muscle and plasma [62,63], and increased utilization [64].

Recent observational data suggested that a large intake of protein (1.2 to 1.5 g/kg per day) was associated with better outcomes in one study and contradictory effects in another [33,34]. In a landmark study, Ishibashi and colleagues [65] showed that 1.5 g/kg per day was associated with the least negative total body protein balance. A protein dosing trial has recently been completed, but until the results are available and in the absence of high-quality prospective trials designed to specifically address the issue of optimal protein intake [66], data from the large interventional trials using supplemental parenteral nutrition can be used to try and provide some answers. *Post hoc* analyses of the results of three recent trials [7,17,24] suggested better outcomes in patients who received less protein.

The discrepancies between the results of clinical studies suggest that there is no fixed energy-to-nitrogen ratio that could be applied in all physiological and pathological conditions. Ambulatory or exercising patients require a higher energy intake in contrast to bedridden or critically ill subjects. Furthermore, inactivity and systemic inflammation can induce or exacerbate anabolic resistance, itself leading to muscle atrophy, increased fat mass, and decreased metabolic rate.

Biomarkers of optimal protein and amino acid intake include whole body or tissue protein balance, circulating protein or amino acid levels, physiological functions (muscle strength, immune competence, insulin sensitivity, glutathione, and oxidative stress), and ultimately clinical outcome. The use of techniques to assess lean tissue by ultrasound [67] or computed tomography scan [68] could help to more accurately tailor the amount of protein, but this needs to be studied further.

### Micronutrient requirements

The European critical care population is characterized by suboptimal preadmission micronutrient status: the trace elements particularly affected are selenium, iron, and zinc [69,70]. Micronutrients are often overlooked during nutritional assessment and this may result in provision of suboptimal nutrition in ICU patients. Micronutrients, such as zinc, selenium, copper, and vitamins C, E, and B, are involved in various metabolic processes, either acting as catalysts or facilitating various enzymatic functions. Micronutrient deficiency can result from pre-existing malnutrition, severity of current illness, and adverse effects of therapeutic regimens or procedures. Several critical care conditions and therapies worsen this precarious status with micronutrient-containing biological losses, such as major burns, major trauma, pathological intestinal losses, and during continuous renal replacement therapy. The inflammatory response further causes a redistribution of micronutrients from the circulating compartment to organs involved in acute phase-related synthetic mechanisms [71]. Confronted by an elevated oxidative stress, patients are not able to develop normal antioxidant and immune defenses.

## Consequences of inappropriate feeding

### Underfeeding

Observational studies have shown the association between negative energy balance and poor outcome [29-32]. Heyland and colleagues [32] showed that the best survival was observed when calorie intake was around 80% of the prescribed target. Recent prospective randomized controlled trials (RCTs) have been criticized for comparing underfed with very underfed patients [72,73] or for overfeeding patients [7,24]. The controversial issues related to energy requirements were discussed earlier in the dedicated section.

### Re-feeding

The re-feeding syndrome is the result of re-initiation of enteral or parenteral feeding in a previously malnourished patient. Complications of this syndrome include electrolyte abnormalities (hypophosphatemia, hypokalemia, and hypomagnesemia) along with sodium and fluid retention potentially leading to heart failure, respiratory failure, and death. Severe hypophosphatemia, in particular, is an early warning sign, and serum phosphate levels should be closely monitored in patients at risk of the re-feeding syndrome.

Starvation for a period as short as 48 hours and poor nutritional status can already predispose to the re-feeding syndrome. Patients at risk should be fed slowly, and electrolyte and other micronutrient levels should be closely monitored and supplemented as required [74]. In contrast to general recommendations to slowly increase calorie intake in malnourished patients to prevent a re-feeding syndrome, several RCTs have demonstrated reduced mortality with early initiation of enteral nutrition [75]. It is likely that many patients are malnourished as a result of prolonged starvation before ICU admission. Therefore, it is unclear whether ICU patients with risk factors for re-feeding syndrome can tolerate more aggressive nutritional support while controlling for the possible re-feeding syndrome by providing optimal electrolyte supplementation, controlled fluid balance, and monitoring of organ function. This issue is currently being investigated in a phase II randomized clinical trial (Australian and New Zealand Clinical Trials Registry number 12609001043224).

### Overfeeding

Provision of macronutrients in excess of metabolic demand is deleterious. In critically ill patients, enteral nutrition is frequently associated with underfeeding and intolerance, whereas parenteral nutrition has been associated with a greater risk of infectious complications and overfeeding [7,24,25,76]. Overfeeding may be associated with hypercapnia and re-feeding syndrome [77,78] and may occur in up to 19% of mechanical ventilation days [79]. High doses of protein intake may lead to azotemia, hypertonic dehydration, and metabolic acidosis [25]. High doses of glucose infusion may result in hyperglycemia, hypertriglyceridemia, and hepatic steatosis [80], although these metabolic abnormalities can be prevented to a large extent by insulin treatment targeting normoglycemia [81].

To avoid overfeeding, some advocate measurement of EE using indirect calorimetry [28]. However, as discussed earlier, the optimal amount of energy that should be administered to ICU patients is not yet determined. In addition, caloric needs may change during the ICU stay, increasing the difficulties of determining the exact amount of calories to prescribe [28]. If such monitoring is unavailable, a feeding protocol may limit the risk of overfeeding [82].

### Autophagy

Insufficient autophagy in prolonged critical illness may cause inadequate removal of damaged proteins and mitochondria [83]. Incomplete clearance of cellular damage, inflicted by illness and aggravated by hyperglycemia, possibly explains the lack of recovery from organ failure in patients with prolonged critical illness and provides potential perspectives for therapies that activate autophagy [83]. In animal experiments, impaired core autophagy machinery may, in concert with downregulated chaperone expression and protein synthesis, collectively affect the proteostasis in skeletal muscle and exacerbate disease progression in critical illness myopathy [84]. Administration of parenteral nutrients, in particular protein- and lipid-enriched parenteral feeding rather than glucose, in the early phase of critical illness has been shown to suppress autophagy in vital organs and muscle and to increase the accumulation of damaged mitochondria and toxic protein aggregates [47]. In humans, such suppression of autophagy with early parenteral nutrition was also shown to increase muscle weakness and to impair recovery thereof [48]. Whether activation of autophagy, using synthetic pharmacological agents or glutamine, as shown in an animal model of critical illness [85], will have therapeutic potential in patients remains to be investigated.

## Pharmaconutrition and immunonutrition

The concept of ‘immune-enhancing formulas’ or ‘immunonutrition’ has been used to characterize solutions enriched with several different nutrients thought to boost the immune response, whereas ‘pharmaconutrition’ was more recently introduced to define the addition of any specific nutrient to a standard formula, at any dose. Although these concepts have exciting implications, their importance remains controversial. Studies have shown that various nutrients have effects on the immune system, metabolism, and gastrointestinal structure and function. Such nutrients may be macronutrients that exert specific effects, such as the amino acids glutamine and arginine or lipids like omega-3 fatty acids; they may also be micronutrients, such as antioxidant vitamins A, C, and E and the minerals selenium and zinc. These pharmaconutrients have been added to commercially available products to produce so-called ‘immunonutrition’ and ‘immune-modulating’ or ‘immune-enhanced’ diets. These solutions have been tested in a number of RCTs to evaluate their impact in critically ill patients. The largest study, which included 597 patients with different underlying diseases, showed that a high-protein formula enriched with arginine, glutamine, antioxidants, and omega-3 fatty acids had no significant effect on outcome [86]. Hence, current evidence does not support the use of pharmaconutrients [53]. However, the need for each pharmaconutrient should be assessed separately, as the risk-to-benefit ratio will be different according to the clinical circumstances, doses, timing, and type of compound.

### Arginine

Arginine stimulates hormonal release and can be metabolized through a family of NO synthase enzymes to nitrogenous compounds like NO. There is a delicate balance of NO levels in critically ill patients. In disease states in which inducible NO synthase is upregulated, NO production can become excessive and can cause harm in terms of hemodynamic instability, immunologic dysfunction, and non-specific cytotoxicity. Arginine administration may therefore be deleterious in critically ill patients [87]. On the other hand, arginine depletion may occur after surgery, even in well-nourished patients. In an RCT in non-critically ill patients with gastrointestinal cancer, preoperative oral supplementation with a specialized diet, including extra L-arginine, was associated with a significantly lower incidence of postoperative infections and reduced length of hospital stay compared with the conventional group [88]. A recent meta-analysis of 32 RCTs showed that 5 days of preoperative arginine and fish oil supplementation reduced the incidence of postoperative infections in non-critically ill patient populations [89].

### Glutamine

Critically ill patients often have decreased glutamine levels on ICU admission, and low plasma glutamine levels are associated with increased mortality [62]. Glutamine administration may improve gut barrier function as well as lymphocyte function, and this could potentially reduce infectious complications. Administration of glutamine as a nitrogen donor for glutathione synthesis may also help to preserve lean body mass and it is an important antioxidant. Several small early studies suggested that enteral glutamine supplementation could reduce infectious complications in critically ill patients [90,91]. However, more recent studies, using parenteral administration, have given conflicting results. These various studies compared very different approaches in both dosing and timing, had different rationales and physiological backgrounds, and asked different questions; they do not, therefore, necessarily represent different sides of a controversy.

In the Scottish Intensive Care Glutamine or Selenium Evaluative Trial study [92], parenteral administration of glutamine was not associated with any measurable improvement in new infection rates or survival. In contrast, the Scandinavian glutamine trial [93] indicated a significant reduction in mortality in the per-protocol analysis of patients who received glutamine for more than 3 days. Recently, Rodas and colleagues [63] suggested that not only low admission plasma glutamine levels but also high levels of more than 930 μmol/L were associated with poor outcome. A recent meta-regression analysis of temporal trends in mortality in patients given parenteral glutamine supplementation or controls not receiving this supplementation showed that the beneficial effects of glutamine on mortality have decreased over the last 20 years [94]. Another meta-analysis of RCTs concluded that publication bias may have explained the reduced rate of infections reported in some of the studies [95]. The most recent meta-analysis of RCTs of parenterally administered glutamine supplementation, that did not include the (Reducing Deaths due to Oxidative Stress (REDOXS) trial [96], reported that parenteral glutamine supplementation combined with nutrition support was associated with reduced hospital mortality and length of stay [95].

The recent REDOXS trial [96] showed a dramatic increase in mortality rates with high doses of enteral and parenteral glutamine (0.6 g/kg per day). Even though there were more patients with three or more organ systems (including renal failure) failing in the glutamine group than in the control group [97], a strong trend toward increased mortality with glutamine remained after adjustment for this imbalance [98,99]. In another study, high-protein enteral nutrition enriched with glutamine and ‘immune-modulating nutrients’ did not reduce infectious complications or improve other clinical endpoints versus standard high-protein enteral nutrition and may have been harmful as suggested by an increased adjusted mortality at 6 months [100]. Therefore, the use of glutamine in ICU patients should be considered with caution until the mechanisms behind the harmful effects reported in the REDOXS study are better understood [101-103].

### Omega-3 fatty acids

The ratio of omega-6 to -3 was 0.8:1.0 in the paleolithic human diet but is 15 to 16.7:1.0 in the present US diet. The anti-inflammatory effects of immune-modulating enteral diets with fish oils have been tested in patients with acute lung injury and acute respiratory distress syndrome. A meta-analysis indicated a 60% mortality reduction when omega-3 fatty acids were administered continuously with full enteral nutrition [104]. However, recent meta-analyses including the latest studies do not confirm such benefit [105,106]. The mode of administration of fish oil, the composition of the control solution, and the differing incidences of diarrhea, suggesting differences in absorption, have been proposed to explain some of the discrepancies in the results of clinical studies. Alternatively, the divergent results may suggest that pharmaconutrients should be given as part of complete nutrition or not at all.

Older retrospective studies reported dose-dependent improvements in outcome of patients receiving intravenous omega-3 fatty acids [107]. Unfortunately, the paucity of data and the poor methodological quality of the available trials do not allow a recommendation regarding the use of parenteral fish oil-based solutions [108,109]. A recent large double-blind randomized clinical trial comparing soybean oil-based versus olive oil-based lipid emulsions failed to demonstrate any difference in outcome between the two solutions [110].

### Micronutrients

Micronutrient deficiency can impair immunity, wound healing, and organ function and is associated with increased oxidative stress with increased concentrations of ROS, which can be overcome by the administration of high doses of trace elements [111]. Two concepts prevail in the literature: (1) replacement of losses (from an acute deficiency condition) with doses remaining within 10 to 15 times the recommended nutritional intake; these losses have been associated with improved immune response, reduction of infectious complications, improved wound healing, and reduction of hospital stay [112-114]; and (2) supplementation with doses 20 to 50 times above nutritional doses in patients with sepsis or respiratory failure or both [115].

Despite controversy regarding optimal doses, meta-analyses have repeatedly shown benefits on mortality and infections of these studies [116-118], most trials having been conducted in European populations. The largest prospective trial did not demonstrate any effect of antioxidant supplementation instituted early in patients with at least two organ failures (including renal failure) [96], a finding that is probably explained by the absence of selenium deficit in the North American population related to the high soil selenium content. Hence, data from recent large-scale studies [96,100] do not support the use of supplemental selenium or vitamins in heterogeneous populations of critically ill patients, as no improvement in outcome was associated with these interventions, in contrast with previous data in specific patient groups (see [119] for a detailed discussion of this issue).

### Pre-, pro- and synbiotics

The World Health Organization defines probiotics as ‘live microorganisms, which, when administered in adequate amounts, confer a health benefit on the host’ [120]. Prebiotics are basically food for probiotics and are non-digestible by humans and stimulate the growth of so-called beneficial bacteria. Common prebiotics are inulin and carbohydrate fibers (oligosaccharides). A synbiotic is a supplement that contains both probiotics and prebiotics.

Critical illness results in changes to the microbiology of the gastrointestinal tract, leading to a loss of commensal flora and an overgrowth of potentially pathogenic bacteria. Administering certain strains of probiotics to critically ill patients may restore a balanced microbiota and have positive effects on immune function and gastrointestinal structure and function. Theoretical risks of transfer of antibiotic-resistance genes from *Lactobacillus* strains resistant to vancomycin to more pathogenic organisms, particularly *Enterococci* and *Staphylococcus aureus*, are possible but have not been established. Translocation resulting in iatrogenic infection has been reported only in case reports and has uniformly occurred in individuals with particular risk factors, such as uncontrolled diabetes and endovascular prostheses. Safety concerns emerged after publication of the Probiotics in Pancreatitis Trial [121], which showed increased mortality from gut ischemia in the probiotic-treated group. However, significant protocol violations, ethical concerns, and the use of a post-pyloric route for a fiber-containing formula limit the external validity of this trial.

The US Food and Drug Administration has clarified that their limited review of probiotics as a dietary supplement applies only to consumption by healthy people and that any use of probiotics to prevent, treat, or mitigate disease would define probiotics as a ‘drug’.

Although all trials performed to assess the effects of probiotics during acute illness were included, no risk of adverse event was found. A recent meta-analysis of 13 RCTs including 1,439 patients demonstrated that probiotic administration did not significantly reduce duration of mechanical ventilation or ICU or hospital mortality rates but did reduce the incidence of ICU-acquired pneumonia and length of ICU stay [122]. A meta-analysis comprising data from more than 11,000 patients showed that probiotics significantly reduced antibiotic-associated diarrhea in all types of patients [123]. Despite these results, concerns remain related to the identification of which critically ill patients could benefit from this approach.

## Early enteral nutrition

The concept of EEN, defined as enteral nutrition initiated within 24 hours after admission, has been adopted by many ICUs on the basis of its positive influence on gut barrier function, increasing secretion of mucus, bile, and immunoglobulin and favorable effects on gut-associated/mucosa-associated lymphoid tissue, release of incretins and other entero-hormones that have a major effect on intermediary metabolism, gut function, and hepatic functions, and its significant effects on morbidity and mortality in RCTs including a total of less than 300 patients [124]. In stable patients on vasopressors, EEN commenced after initial resuscitation appears to be safe and confers a survival benefit [75,125]. Several independent meta-analyses have confirmed a better outcome in patients receiving EEN compared with patients not receiving EEN, even though methodological deficiencies were found for some studies [126].

Some ICU patients receiving enteral nutrition may present clinical signs of intolerance such as increased gastric residual volume (GRV). This problem may be circumvented by the introduction of post-pyloric feeding tubes. Another approach is to accept higher amounts of GRV. The optimal approach is still controversial. A recent systematic review identified six RCTs and six prospective observational studies analyzing different thresholds of GRV to guide enteral nutrition and to prevent complications (for example, vomiting, aspiration, and nosocomial pneumonia) in mechanically ventilated patients [127]. Because of the heterogeneity in outcome measures, patient populations, types and diameters of feeding tubes, and randomization procedures, a formal meta-analysis was not appropriate. Analysis of high-quality RCTs in medical patients could not demonstrate an association between complication rate and the magnitude of GRV. The authors concluded that monitoring of GRV appears unnecessary to guide nutrition in mechanically ventilated patients with a medical diagnosis. Because one observational study [128] suggested an increased frequency of aspiration if a GRV of more than 200 mL was registered more than once, surgical patients may benefit from a lower GRV threshold (200 mL). Another recent study [129] reported that not measuring GRV in medical ICU patients was associated with an increase in nutritional intake without additional risk of aspiration pneumonia.

## Conclusions

Well-established beliefs in the metabolic and nutritional fields of critical illness have been challenged by recent findings from large-scale, prospective RCTs. The numerous uncertainties and unresolved issues unraveled by these recent studies and outlined in Table 1 highlight the urgent need for more basic and clinical research on this important topic. For daily clinical practice, awareness of the controversial issues as well as of the areas of consensus (Table 2) is needed. We hope that this article can help clinicians understand that take-home messages are difficult to draw when based on conflicting evidence. We wrote this article to underline priorities for research in order to be able to provide more robust evidence to support recommendations for clinical practice. Meanwhile, updated recommendations, even weak ones, represent the best tool to guide intensivists through the growing number of uncertainties.

**Table 1:** **Areas of uncertainty – opposing views**

**Topic/area**	**One viewpoint**	**Opposing view**
Optimal caloric intake	Early match of EE.	Less than EE during the early phase.
Supplemental PN	When EN provision is less than 60% in early course of ICU stay not contraindicated.	Not before day 8 in patients with a body mass index of at least 17.
Optimal protein intake	Equal to nitrogen losses, up to 1.5 g/kg per day.	Less than nitrogen losses.
Re-feeding syndrome	Slowly increase nutritional support to prevent re-feeding syndrome consequences even if this results in increased energy deficit.	Early nutritional support improves outcome also in malnourished patients; re-feeding syndrome consequences should be monitored and immediately treated if necessary.
Role of indirect calorimetry	Yes (patients staying more than 4 days).	No.
Autophagy	Provision of nutrients should be reduced so as not to reduce autophagy capacity as early nutrients provoke a phenotype of suppressed autophagy in human and animal experiments, with functional consequences that impair recovery.	Although experimentally autophagy may be reduced in early critical illness, pharmacological autophagy activation remains to be tested clinically.
Antioxidants	Supplement in case of low levels of antioxidants.	Use pharmacological dosages.
Glutamine	In all patients on PN.	High-dose glutamine increases mortality in critically ill patients, regardless of route of administration.
Omega-3 lipid formulations	Use continuous enteral administration and avoid bolus administration.	Not beneficial in acute respiratory distress syndrome.
High-dose selenium 800 to 4,000 μg/day	High-dose trials (1,000 μg) show greater improvement than low-dose trials.	Potential for toxicity.
		In selenium-replete populations, 800 to 1,000 μg may be ineffective.
Probiotics	Safe. Avoid use in pancreatitis patients with multiple organ dysfunction syndrome.	May be harmful in ICU patients when given post-pyloric with fiber.
Monitoring GRV	Accept GRV of 250 up to 500 mL per 6 hours.	Abandon GRV monitoring in medical patients and consider in surgical patients.

EE, energy expenditure; EN, enteral nutrition; GRV, gastric residual volume; PN, parenteral nutrition.

**Table 2:** **Areas of consensus (ICU patients with a more than 4-day length of stay)**

	**Consensus**
Early enteral feeding	Consider in each patient without absolute contraindication; prevents mucosal atrophy
Risks of overfeeding	Early phase
Estimation of energy expenditure	Requires indirect calorimetry – cannot be predicted by equations
Arginine	Not recommended in sepsis; beneficial in perioperative patients outside the ICU
Vitamins, trace elements	Mandatory, in nutritional doses; particularly true in parenteral nutrition

## Abbreviations

EE: Energy expenditure
EEN: Early enteral nutrition
EPaNIC: Impact of early parenteral nutrition completing enteral nutrition in adult critically ill patients
GRV: Gastric residual volume
IIT: Intensive insulin therapy
NO: Nitric oxide
RCT: Randomized controlled trial
REDOXS: Reducing deaths due to oxidative stress
ROS: Reactive oxygen species
